# Dissecting the Genetic Basis of Grain Size and Weight in Barley (*Hordeum vulgare* L.) by QTL and Comparative Genetic Analyses

**DOI:** 10.3389/fpls.2019.00469

**Published:** 2019-04-24

**Authors:** Qifei Wang, Genlou Sun, Xifeng Ren, Binbin Du, Yun Cheng, Yixiang Wang, Chengdao Li, Dongfa Sun

**Affiliations:** ^1^College of Plant Science and Technology, Huazhong Agricultural University, Wuhan, China; ^2^Department of Biology, Saint Mary’s University, Halifax, NS, Canada; ^3^School of Veterinary and Life Sciences, Murdoch University, Murdoch, WA, Australia; ^4^Hubei Collaborative Innovation Centre for Grain Industry, Yangtze University, Jingzhou, China

**Keywords:** barley (*Hordeum vulgare* L.), grain size and weight, doubled haploid population, comparative genomics, QTL

## Abstract

Grain size and weight are crucial components of barley yield and quality and are the target characteristics of domestication and modern breeding. Despite this, little is known about the genetic and molecular mechanisms of grain size and weight in barley. Here, we evaluated nine traits determining grain size and weight, including thousand grain weight (Tgw), grain length (Gl), grain width (Gw), grain length-width ratio (Lwr), grain area (Ga), grain perimeter (Gp), grain diameter (Gd), grain roundness (Gr), and factor form density (Ffd), in a double haploid (DH) population for three consecutive years. Using five mapping methods, we successfully identified 60 reliable QTLs and 27 hotspot regions that distributed on all chromosomes except 6H which controls the nine traits of grain size and weight. Moreover, we also identified 164 barley orthologs of 112 grain size/weight genes from rice, maize, wheat and 38 barley genes that affect grain yield. A total of 45 barley genes or orthologs were identified as potential candidate genes for barley grain size and weight, including 12, 20, 9, and 4 genes or orthologs for barley, rice, maize, and wheat, respectively. Importantly, 20 of them were located in the 14 QTL hotspot regions on chromosome 1H, 2H, 3H, 5H, and 7H, which controls barley grain size and weight. These results indicated that grain size/weight genes of other cereal species might have the same or similar functions in barley. Our findings provide new insights into the understanding of the genetic basis of grain size and weight in barley, and new information to facilitate high-yield breeding in barley. The function of these potential candidate genes identified in this study are worth exploring and studying in detail.

## Introduction

Since domestication about 10,000 years ago in the Fertile Crescent, barley (*Hordeum vulgare* L.) has become one of the most important cereal crops cultivated around the world, and is widely used as animal feed, potential healthy food products and is a major raw material for malting and brewing industries (Salamini et al., 2002; Collins et al., 2010; Ullrich, 2010). With the rapid growth of the global population and the continuous reduction of arable land worldwide, improving barley yield remains a major challenge for the barley breeding program in the present context of climate change (Fischer and Edmeades, 2010; Feng et al., 2016).

Grain size and weight, which is determined by its three-dimensional structure (length, width, and thickness) and the degree of grain filling, are two crucial components that affect barley yield and malt quality (Zhang X. et al., 2012). In the context of crop domestication and artificial breeding, grain size and weight have always been important agronomic traits for human care and selection. Despite this, little is known about the genetic and molecular mechanisms of grain size and weight in barley. Archaeological evidence suggests that barley grains increased in size starting in the Pre-Pottery Neolithic A (PPNA; 9700–8700 BC) and earliest Pre-Pottery Neolithic B (PPNB; 8700–6200 BC) (Fuller, 2007). Compared to their progenitors, modern barley varieties have larger grains that not only have a favorable effect on seedling vigor and yield, but are also favored by the malting and feed industries as they can increase malt yields and feed production capacity (Gan and Stobbe, 1996; Walker and Panozzo, 2011). Therefore, untangling the genetic factors controlling grain size and weight is crucial for improving barley yield and quality as well as understanding the domestication process that has occurred in barley.

In recent years, the rapid advance of functional genomics research has promoted our understanding of the genetic basis and developmental mechanisms of grain size and weight, many QTLs or genes associated with grain size and weight have been mapped or characterized in detail in rice (Zuo and Li, 2014; Li and Yang, 2017; Liu et al., 2017a, 2018; Wu et al., 2017; Li et al., 2018; Sun et al., 2018; Ying et al., 2018; Zhao et al., 2018), maize (Liu et al., 2015, 2017b; Chen et al., 2016; Zhou et al., 2017), and wheat (Zhang L. et al., 2012; Hu et al., 2016; Ma L. et al., 2016; Ma M. et al., 2016; Geng et al., 2017; Hou et al., 2017; Sajjad et al., 2017; Zhai et al., 2018; Zhang et al., 2018). However, in comparison to the relatively extensive research that has been conducted in other cereal species mentioned above, only limited molecular information is available to understand the biological developmental processes and formation mechanism of barley grain size. To date, some attempts have been made to clarify the genetic basis of barley grain size and weight. For example, Ayoub et al. (2002) detected QTLs for grain size and shape characteristics on all seven linkage groups. Walker et al. (2013) identified 232 QTLs for 11 grain traits across the three environments in a DH population. Zhou et al. (2016) mapped two major QTLs for grain length in a recombinant inbred line (RIL) population. Xu et al. (2018) identified 29 QTL hotspots distributed on all seven chromosomes for grain size and weight. Additionally, several genes affecting barley grain size or weight have been characterized using mutant materials, including *Nud* (Taketa et al., 2008), *Vrs1* (Komatsuda et al., 2007; Sakuma et al., 2017), *Vrs2* (Youssef et al., 2017), *Vrs3* (Bull et al., 2017; Van Esse et al., 2017)*, Vrs4* (Koppolu et al., 2013), and *Int-c* (Ramsay et al., 2011). However, these genes that have been characterized in the past all indirectly affect barley grain size or weight, while genes that directly control yield components have not yet been identified in barley.

Comparative genomic approaches have provided an effective strategy for identifying genes with conserved functions across genomes and species (Su et al., 2011; Liu et al., 2017b), such as wheat powdery mildew resistance gene *TmMla1* and barley powdery mildew resistance gene *HvMLA1*, which were identified as orthologous (Jordan et al., 2011), and the *Int-c* gene regulating lateral spikelet fertility in barley which was identified as an ortholog of the maize domestication gene *ZmTB1* (Ramsay et al., 2011). It has been reported that many of the genes affecting grain size/weight generally have conserved functions, but their functional specificity may be divergent among species (Zhang et al., 2015, 2018; Liu et al., 2017b; Sajjad et al., 2017; Zhai et al., 2018). For example, the *GW2* gene that encodes a RING-type E3 ubiquitin ligase to regulate grain weight in rice (Song et al., 2007), its orthologous in maize (Li et al., 2010), wheat (Su et al., 2011; Bednarek et al., 2012; Yang et al., 2012; Hong et al., 2014; Jaiswal et al., 2015; Simmonds et al., 2016; Geng et al., 2017; Zhai et al., 2018; Zhang et al., 2018), and sorghum (Tao et al., 2017) were also found to be involved in the control of grain weight, but with different mechanisms. Similarly, the orthologous genes of rice *GS5* gene (Li et al., 2011) in maize (Liu et al., 2015) and wheat (Wang et al., 2015, Ma L. et al., 2016; Wang S. et al., 2016) also control similar phenotypes in grain size and weight as well as in rice. At present, a considerable number of genes associated with grain size/weight have been characterized in rice, maize, and wheat, but whether the orthologs of these genes in barley have the same or similar function remains an open question. Therefore, it is necessary to characterize the orthologs of these grain size/weight genes in barley to provide insight into the genetic mechanisms of barley grain size and weight.

In this study, we performed a QTL mapping of nine grain size and weight traits in a DH population, using an SNP-based high density genetic map and identified 164 barley orthologs of 112 grain size/weight genes from rice, maize, and wheat in the barley genome. The objectives of this study were to identify reliable QTL and QTL hotspots affecting barley grain traits, and to explore the genetic correspondence between the QTLs identified here and grain size/weight genes in other cereal species. The results of this study will enhance our understanding of the genetic basis of grain size and weight in barley and may provide new information to facilitate high-yield breeding in barley.

## Materials and Methods

### Plant Materials and Field Trials

A doubled haploid (DH) population containing 122 lines derived from Huaai 11 (six-rowed and naked) and Huadamai 6 (two-rowed and hulled) was employed to identify QTLs that control barley grain size and weight. Details of the DH population and their parents can be found in our earlier studies (Ren et al., 2010; Wang et al., 2017). The DH lines and parents were evaluated in natural field conditions over three crop seasons (2015 to 2016, 2016 to 2017 and 2017 to 2018 seasons) in the experimental farm of Huazhong Agricultural University, Wuhan, China (30°48’N, 114°36’E), in a randomized complete block design with three replicates. In each replicate, each line was planted in a two-row plot of 1.5 m length with the spacing of 0.1 m between plants and 0.2 m between rows. Field management, including irrigation, fertilization, weeding and pest control, followed the standard agricultural practices in barley production.

### Evaluation of Barley Grain Size and Weight

At maturity, kernels of six uniform plants in the middle of each plot were bulk-harvested and sun-dried for phenotypic evaluation. Then, 200 to 300 fully filled grains of each line were used to measure thousand grain weight (Tgw, g), grain length (Gl, mm), grain width (Gw, mm), grain length-width ratio (Lwr), grain area (Ga, mm^2^), grain perimeter (Gp, mm), grain diameter (Gd, mm) and grain roundness (Gr) using a camera-assisted phenotyping system (SC-G, Wanshen Detection Technology Co., Ltd., Hangzhou, China) (Yin et al., 2015). Ga and Gp were defined as the actual area and length of the seed projection outline, respectively. Gd was calculated by Gd = $\sqrt{4\times \mathrm{Ga}/\pi }$, and Gr was calculated by Gr = $\frac{4\times \mathrm{Ga}}{\pi \times {\left(\mathrm{major axis}\right)}^{2}}$, where the major axis is the major axis length of the seed fitted ellipse. In addition, in order to assess difference in grain density, we calculated the factor form density (Ffd, g/mm^2^) according to the following formula: Ffd = $\frac{\mathrm{Tgw}}{1000\times \mathrm{Gl}\times \mathrm{Gw}}$ (Giura and Saulescu, 1996).

### Statistical Analysis

The best linear unbiased prediction (BLUP) value of the three replicate measurements for each year were used for statistical analysis and QTL mapping. The calculations of descriptive statistics, Student’s *t*-test, normality test (Shapiro–Wilk), correlation analysis and analysis of variance (ANOVA) were performed using SPSS v24.0 (IBM SPSS Statistics, Chicago, IL, United States). Broad sense heritability (*H*^2^) estimates were calculated from ANOVA using the following formula: *H*^2^ = 1 − *MS*_2_/*MS*_1_, where *MS*_1_ and *MS*_2_ are the mean squares of genotype and genotype × environment, respectively (Knapp et al., 1985). Frequency distribution and QTL-likelihood maps for the grain size and weight were drawn using the Origin programs (OriginLab, Northampton, MA, United States).

### QTL Analysis

The high-density genetic linkage map for “Huaai 11 × Huadamai 6” population used in this study was constructed previously (Ren et al., 2016), which included 1962 markers on all seven chromosomes, comprising 1894 SNP markers and 68 SSR markers. It spanned 1375.80 cM of the whole-genome with an average marker distance of 0.7 cM. Grain size and weight QTLs detection were performed using the inclusive composite interval mapping (ICIM) algorithm in QTL IciMapping v4.1 software (Meng et al., 2015). Single-environment QTL and Multi-Environment Trials (MET) analyses were performed using the ICIM-ADD (additive and dominance effects) mapping method in “BIP (QTL mapping in biparental populations)” module and “MET (QTL mapping for multi-environment traits)” module, respectively. The scanning step size was 1.0 cM, and the probability in stepwise regression (PIN) was 0.001. The LOD threshold was determined by a 1000 times the permutation test, with a Type 1 error of 0.05. The narrow sense heritability of each MET QTL was estimated from the MET analysis using the following formula: h^2^ = $h^{2}=\frac{\mathrm{PVE}(A)}{\mathrm{PVE}(A)+\mathrm{PVE}(E)}$, where *PVE(A)* and *PVE(E)* are the additive genetic (A) and environmental (E) components of the multi-environment variance. Moreover, to overcome the interference of row type (Rt) and caryopsis type (Ct), we used Rt and Ct as a covariate, respectively, and conducted QTL mapping with a covariance analysis. The covariate QTL analysis was performed using software QTL.gCIMapping from the R website (Feng et al., 2018); the critical LOD scores for a significant QTL was set at 3.0, and the walking speed for the genome-wide scan was set at 1 cM. The naming of QTLs followed the nomenclature proposed by Mccouch et al. (1997). If QTLs for different traits were located in the same marker interval or their 1.5-LOD confidence intervals overlapped, the corresponding loci were defined as a pleiotropic or tightly linked QTLs. For the same trait, QTLs repeatedly detected in more than one year were defined as a stable QTLs, QTLs repeatedly detected in at least two years environment and in multiple mapping methods were considered to be a reliable QTLs. The orthologs of rice, wheat and maize genes in the barley reference genome (Mascher et al., 2017) were identified using the Ensembl Plant Database^1^. BARLEYMAP pipeline (Cantalapiedra et al., 2015)^2^ was used to compare the marker information and identify potential candidate genes based on the barley physical map. According to the marker density of the genetic map, any barley gene or ortholog located within ± 5 Mb on either side of the QTL peak was identified as a candidate gene for QTL.

## Results

### Phenotypic Variation and Correlation Analysis

**FIGURE 1:**
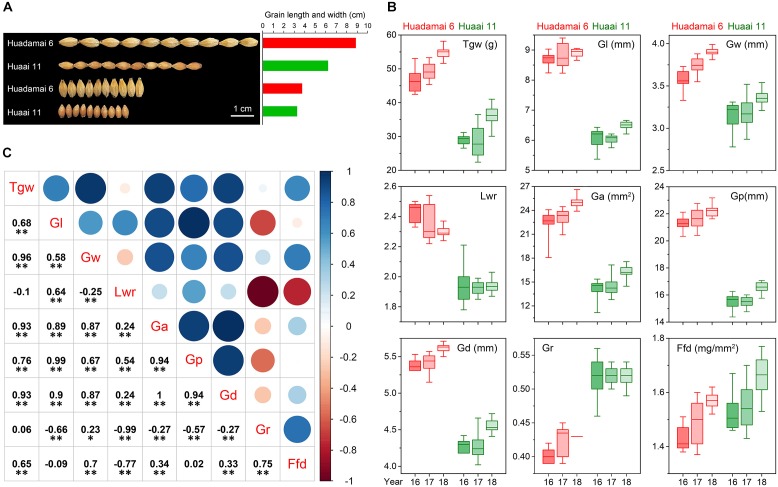
**(A)** Grain phenotypes of the two parents Huadamai 6 and Huaai 11. **(B)** Box diagram of nine grain size and weight traits for two parents in three years. **(C)** Pearson correlation coefficients among nine characteristics of barley grain size across the three years. The two-tailed *t*-test was applied to test the significance of correlation coefficients (^∗^*p* < 0.05, ^∗∗^ *p* < 0.01).

We evaluated nine grain size and weight traits in the DH population and their parents, for three consecutive years. Descriptive statistics for those grain size and weight traits are presented in Table 1. The phenotypic difference in the grain of the two parental lines, Huadamai 6 and Huaai 11, are shown in Figure 1A. The *t*-test showed that two parents were significantly different (*p* < 0.05) on all investigated grain size and weight traits (Table 1). Huadamai 6 showed higher values for Tgw, Gl, Gw, Lwr, Ga, Gp, and Gd in all three years than Huaai 11, while Huaai 11 had more Gr and Ffd than Huadamai 6 (Table 1 and Figure 1B).

**Table 1:** Phenotypic performance for the nine grain size and weight traits of the DH population and their parents.

Trait^a^	Year	Huadamai 6		Huaai 11		*T*-value^c^	DH population						
		Mean	SD^b^	Mean	SD^b^		Max	Min	Mean	SD^b^	CV^d^	Skewness	Kurtosis
Tgw (g)	2016	45.34	2.28	29.99	0.82	21.53^∗∗^	61.86	21.97	36.78	9.64	26.22	0.78	−0.02
	2017	49.24	2.37	29.79	2.72	19.08^∗∗^	60.80	16.89	33.73	11.22	33.25	0.79	−0.40
	2018	54.42	1.15	36.12	2.72	25.99^∗∗^	61.15	19.66	36.61	10.90	29.77	0.64	−0.51
Gl (mm)	2016	8.77	0.13	6.10	0.22	32.01^∗∗^	9.21	5.43	7.22	1.11	15.35	0.08	−1.44
	2017	8.94	0.33	6.07	0.10	30.10^∗∗^	9.76	5.83	7.46	1.07	14.34	0.10	−1.23
	2018	8.89	0.13	6.48	0.13	53.56^∗∗^	9.35	5.88	7.51	1.03	13.68	0.05	−1.28
Gw (mm)	2016	3.62	0.07	3.26	0.04	13.99^∗∗^	3.60	2.33	2.96	0.30	10.11	0.34	−0.59
	2017	3.75	0.08	3.20	0.10	15.40^∗∗^	4.02	2.45	3.23	0.41	12.72	0.37	−0.95
	2018	3.89	0.04	3.35	0.10	20.27^∗∗^	4.08	2.59	3.34	0.38	11.39	0.21	−0.85
Lwr	2016	2.43	0.06	1.89	0.07	19.28^∗∗^	3.15	1.82	2.44	0.32	12.91	−0.12	−0.84
	2017	2.39	0.12	1.91	0.04	13.77^∗∗^	3.21	1.79	2.35	0.31	13.38	0.27	−0.45
	2018	2.29	0.03	1.94	0.05	24.34^∗∗^	2.88	1.77	2.27	0.25	11.07	0.09	−0.64
Ga (mm^2^)	2016	22.95	0.58	14.73	0.38	36.64^∗∗^	22.68	9.77	15.28	3.38	22.09	0.43	−0.54
	2017	23.52	0.65	14.49	0.62	35.48^∗∗^	27.22	10.74	17.63	4.01	22.76	0.56	−0.32
	2018	24.88	0.48	16.29	0.76	38.84^∗∗^	27.00	11.88	18.50	3.97	21.47	0.44	−0.53
Gp (mm)	2016	21.46	0.32	15.65	0.38	38.09^∗∗^	21.93	13.47	17.36	2.47	14.21	0.11	−1.32
	2017	21.88	0.56	15.58	0.30	34.58^∗∗^	23.70	14.21	18.38	2.48	13.52	0.12	−1.04
	2018	22.10	0.30	16.57	0.37	47.73^∗∗^	23.07	14.88	18.66	2.42	12.96	0.07	−1.15
Gd (mm)	2016	5.39	0.07	4.32	0.06	36.64^∗∗^	5.36	3.51	4.37	0.48	11.05	0.24	−0.72
	2017	5.46	0.08	4.28	0.09	34.18^∗∗^	5.87	3.67	4.69	0.53	11.41	0.35	−0.56
	2018	5.61	0.06	4.54	0.11	35.48^∗∗^	5.85	3.86	4.80	0.52	10.82	0.24	−0.69
Gr	2016	0.40	0.01	0.53	0.02	−22.55^∗∗^	0.56	0.31	0.42	0.06	14.74	0.53	−0.66
	2017	0.42	0.02	0.53	0.01	−16.11^∗∗^	0.56	0.32	0.43	0.06	13.84	0.27	−0.79
	2018	0.43	<0.01	0.52	0.01	−26.94^∗∗^	0.57	0.34	0.44	0.05	11.76	0.36	−0.62
Ffd (mg/mm^2^)	2016	1.43	0.05	1.51	0.06	−3.68^∗∗^	2.20	1.26	1.71	0.19	11.39	0.23	−0.25
	2017	1.47	0.06	1.53	0.07	−2.32^∗^	1.80	1.01	1.36	0.19	14.17	0.21	−1.01
	2018	1.57	0.03	1.66	0.07	−4.95^∗∗^	1.81	1.12	1.42	0.17	12.02	0.22	−0.93

*^a^Tgw, thousand grain weight; Gl, grain length; Gw, grain width; Lwr, grain length-width ratio; Ga, grain area; Gp, grain perimeter; Gd, grain diameter; Gr, grain roundness; Ffd, factor form density. ^b^SD, Standard deviation. ^c∗^Significant at the 0.05 probability level; ^∗∗^Significant at the 0.01 probability level. ^d^CV, Coefficient of variation in percent.*

The nine grain size and weight traits in the DH population showed highly phenotypic variation and transgressive segregation (values more extreme than the parental phenotypes) in all years. The phenotypic variation coefficient ranged from 10.11 to 33.25% (Table 1). Variance analysis indicated that the effects of genotype, year and genotype × year interactions were all significant (Table 2). All grain size and weight traits had broad-sense heritability over 95%, which confirmed that genetic effects are the major determinant of the phenotypic variance on grain size and weight in barley. The frequency distributions of nine grain size and weight traits showed continuous variation in all years, indicating the polygenic inheritance (Supplementary Figure S1). Shapiro–Wilk test indicated that some of the grain size and weight traits displayed normal distributions, including Lwr (2016–2018) and Ffd (2016).

**Table 2:** Mean squares of ANOVA and heritability for grain size and weight of the DH population in three years.

Source of variation	df	Tgw	Gl	Gw	Lwr	Ga	Gp	Gd	Gr	Ffd
Year	2	1074.26^∗∗^	8.89^∗∗^	14.09^∗∗^	2.63^∗∗^	1012.68^∗∗^	171.68^∗∗^	18.28^∗∗^	0.07^∗∗^	12.61^∗∗^
Genotype	121	977.79^∗∗^	10.07^∗∗^	1.14^∗∗^	0.74^∗∗^	125.88^∗∗^	53.23^∗∗^	2.30^∗∗^	0.03^∗∗^	0.29^∗∗^
Genotype × Year	242	17.44^∗∗^	0.11^∗∗^	0.04^∗∗^	0.03^∗∗^	1.98^∗∗^	0.54^∗∗^	0.03^∗∗^	<0.01^∗∗^	0.01^∗∗^
Error	732	5.56	0.06	0.01	0.01	0.56	0.25	0.01	<0.01	0.01
Heritability (%)		98.22	98.94	96.95	96.61	98.43	98.99	98.52	97.56	96.06

*^∗∗^Significant at the 0.01 probability level.*

Additionally, we also calculated the correlation coefficient among the nine grain size and weight traits in the DH population based on the mean value of the three-year data (Figure 1C). The results showed that Tgw had a significantly positive correlation with all grain size and weight traits except Gr and Lwr. Ffd was highly positively correlated with Tgw, Gw, Ga, Gd and Gr, and was negatively correlated with Lwr. Other seven grain size and weight traits (Gl, Gw, Lwr, Ga, Gp, Gd and Gr) also showed a highly significant correlation with each other.

### QTL Analysis of Barley Grain Size and Weight Trait

**FIGURE 2:**
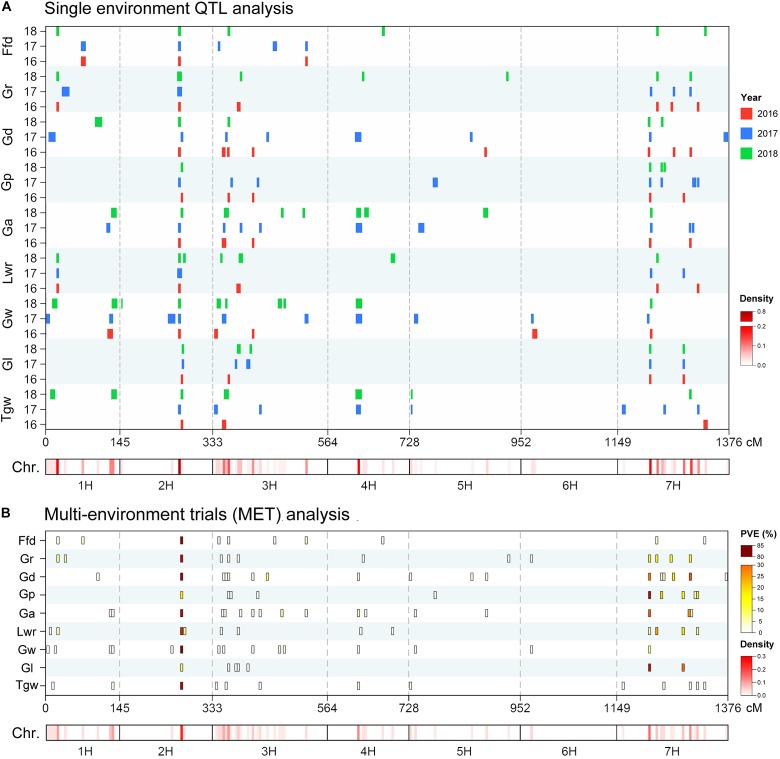
Chromosome distribution of QTLs associated with nine grain size and weight traits identified in the **(A)** single-environment QTL analysis and **(B)** multi-environment trials (MET) analysis. Heat map under the *X*-axis showed the density of QTLs for nine grain size and weight traits across the genome. The window size was 5 cM. QTL bars in single-environment QTL analysis represented the 1.5-LOD support intervals from ICIM mapping.

**FIGURE 3:**
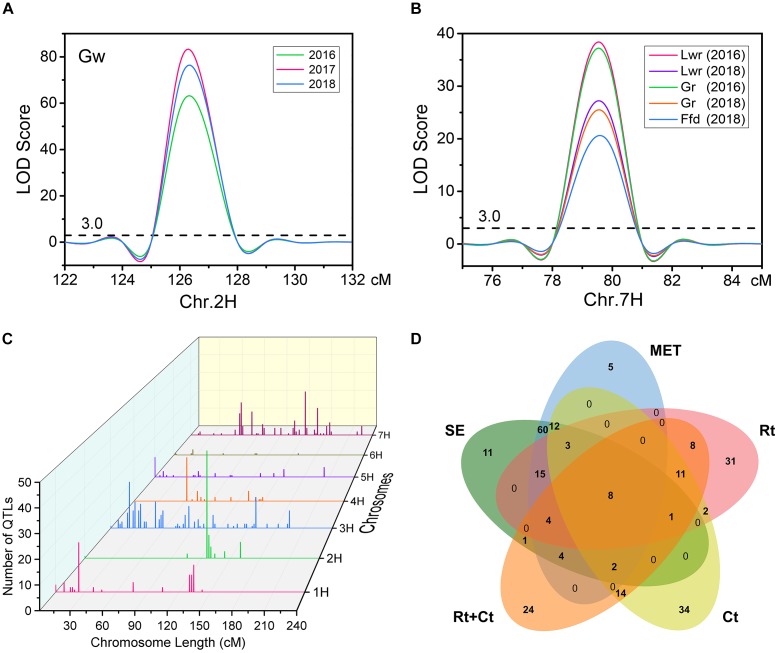
**(A)** A stable QTL on chromosome 2H for Gw was identified in all three years using single environmental QTL analysis. **(B)** A pleiotropic or tightly linked QTL on chromosome 7H was identified for Lwr, Gr, and Ffd in single environmental QTL analysis. **(C)** Distribution characteristics of QTLs for nine grain size and weight traits detected in the five mapping methods. **(D)** Venn diagram of QTLs identified for nine grain size and weight traits in five mapping methods. SE, single-environment QTL analysis; MET, multi-environment trials (MET) analysis; Rt, covariate QTL analysis using row type as a covariate; Ct, covariate QTL analysis using caryopsis type as a covariate; Rt+Ct, covariate QTL analysis using row type and caryopsis type as a covariate.

Using the ICIM BIP module for single-environment QTL analysis, we identified 168 QTLs distributed on all chromosomes of barley for nine grain size and weight traits, including 45, 63 and 60 QTLs in 2016, 2017 and 2018, respectively (Supplementary Table S1 and Figure 2A). Out of the 168 identified QTLs, 62 (36.9%) major QTLs (QTLs that can explaining more than 10% of the phenotypic variation) were identified, including 3, 8, 5, 9, 8, 9, 8, 8, and 4 QTLs for Tgw, Gl, Gw, Lwr, Ga, Gp, Gd, Gr, and Ffd, respectively (Supplementary Table S1). Importantly, 3, 3, 5, 4, 5, 3, 3, 4, and 3 stable QTLs (QTLs repeatedly detected in more than one year for the same trait) were identified for Tgw, Gl, Gw, Lwr, Ga, Gp, Gd, Gr, and Ffd, respectively (Supplementary Table S2). Nineteen stable QTLs each explained more than 10% of the phenotypic variation (mean value from all years). The QTL for Gw around 125 cM on chromosomal 2H, identified in all three years, is shown in Figure 3A as an example. Additionally, we also identified 23 pleiotropic or tightly linked QTLs that influenced at least two traits, such as the QTL at 79.5–80.5 cM (511.81–514.43 Mb) on chromosomal 7H simultaneously affected Lwr, Gr, and Ffd (Supplementary Table S3 and Figure 3B).

We also performed MET analysis using the ICIM MET module. A total of 12, 7, 17, 10, 17, 11, 17, 12, and 10 MET QTLs were identified for Tgw, Gl, Gw, Lwr, Ga, Gp, Gd, Gr, and Ffd, respectively (Supplementary Table S4 and Figure 2B). Of these, 25 (22.1%) MET QTLs each explained more than 10% of the phenotypic variation. The narrow sense heritability of each MET QTL ranged from 24.99 to 99.98%. In addition, we also identified 23 pleiotropic or tightly linked QTLs that affected two or more traits simultaneously (Supplementary Table S5). Notably, a considerable number of loci were repeatedly identified by single-environment QTL analysis and MET analysis. A total of 109 QTLs (96.5%) detected in MET analysis were also identified in a single-environment QTL analysis (Supplementary Table S4). Similarly, 159 QTLs (94.6%) detected in single-environment QTL analysis were also identified in the MET analysis (Supplementary Table S1). In addition, we also found four QTLs were only identified in MET analysis with a low level of explanation of phenotypic variation (Supplementary Table S4).

Furthermore, to eliminate the potential confounding effect of row type (Rt) and caryopsis type (Ct) on grain size and weight traits, we used these two traits as a covariate, and performed covariate QTL analysis using genome-wide composite interval mapping (GCIM) methods. A total of 312 covariate QTLs distributed on all seven chromosomes were successfully identified, including 108, 117, and 87 covariate QTLs for using Rt, Ct, and Rt+Ct as a covariate, respectively (Supplementary Table S6). Among these 312 covariate QTLs, 123 covariate QTLs corresponded to main or MET QTLs detected in single-environment QTL analysis or MET analysis, and the remaining 189 (60.6%) were new QTLs that were not identified in either single-environment QTL analysis or MET analysis (Supplementary Table S6). We integrated all QTLs detected by the five mapping methods and found that most QTLs were distributed on chromosomes 1H, 2H, 3H, and 7H (Figure 3C). Figure 3D shows the Venn diagram of the QTLs for nine grain size and weight traits identified by five mapping methods. Importantly, we identified 60 reliable QTLs (QTLs of the same trait repeatedly detected in at least two years environment and in multiple mapping methods), including 7, 8, 9, 7, 9, 6, 6, 4, and 4 reliable QTLs for Tgw, Gl, Gw, Lwr, Ga, Gp, Gd, Gr, and Ffd, respectively (Table 3). The phenotypic variation explained by each reliable QTL ranged from 0.75 to 70.03% (mean value from all QTLs), with a LOD value ranging from 3.42 to 80.98 (mean value from all QTLs). Among these reliable QTLs, most reliable QTLs were identified in single-environment QTL analysis or MET analysis, only 8 reliable QTLs were only identified in covariance QTL analysis. Importantly, 21 of reliable QTLs had major effects on their respective target traits, of which 20 QTLs were also major QTLs detected in single-environment and multi-environmental.

**Table 3:** Reliable QTLs identified for nine grain size and weight traits in two or more year using multiple mapping method.

Trait	QTL ^a^	Chr. ^b^	Physical interval (Mb)	LOD	PVE ^c^	Add ^d^	Year	Mapping method ^e^	Candidate genes or orthologs
Tgw	***qTgw2-1***	2H	647.83–653.98	3.83–70.02	43.46–80.62	+	2016, 2017, 2018	SE, MET, Ct	*Vrs1/Int-d*
	*qTgw3-2*	3H	660.74–664.33	3.22–5.45	1.42–2.23	+	2016, 2017	SE, Rt, Ct, Rt+Ct	*HvOsBDG1*
	*qTgw4-1*	4H	529.57–532.00	4.76–14.91	1.41–3.00	−	2017, 2018	SE, MET, Rt, Ct, Rt+Ct	
	*qTgw5-1*	5H	0.43–2.57	3.62–10.64	0.87–2.23	−	2016, 2017, 2018	SE, MET, Rt, Ct, Rt+Ct	
	*qTgw7-1*	7H	638.53–639.84	3.12–4.73	0.85–0.99	+	2016, 2017	SE, MET, Rt	*HvGW6a*
	*qTgw7-3*	7H	345.67–381.62	9.36–9.92	1.82–6.55	+	2016, 2018	SE, MET, Rt	*btwd1*
	*qTgw7-5*	7H	79.49–87.38	3.14–5.40	1.05–2.53	+	2016, 2018	SE, MET, Ct, Rt+Ct	
Gl	*qGl2-1*	2H	647.83–653.98	9.36–59.59	2.84–13.19	+	2016, 2017, 2018	SE, MET, Ct	*Vrs1/Int-d*
	*qcGl2-3*	2H	659.60–659.72	3.14–4.05	0.95–1.05	+	2016, 2018	Ct, Rt+Ct	*HvGS2-3*
	*qGl3-2*	3H	604.52–606.01	5.54–10.08	0.70–2.73	+	2017, 2018	SE, MET, Rt, Ct, Rt+Ct	*HvAUX1*
	*qcGl3-9*	3H	285.88–303.30	3.63–10.75	0.52–10.79	−	2017, 2018	Rt, Ct, Rt+Ct	
	***qGl7-1***	7H	546.66–562.79	14.01–170.39	14.71–51.09	+	2016, 2017, 2018	SE, MET, Rt	*Nud; HvTaMOC1*
	***qGl7-2***	7H	382.25–410.75	43.09–131.82	26.17–32.55	−	2016, 2017, 2018	SE, MET	
	*qcGl7-7*	7H	166.04–169.40	4.55–10.56	4.63–7.48	+	2016, 2017, 2018	Rt, Rt+Ct	
	*qcGl7-8*	7H	103.21–109.54	3.50–5.49	1.35–2.33	+	2016, 2017	Ct, Rt+Ct	
Gw	*qGw1-1*	1H	478.21–478.91	3.22–6.24	0.60–1.05	−	2016, 2017	SE, MET, Ct	
	*qGw1-3*	1H	21.61–22.26	3.79–7.61	0.69–1.41	−	2016, 2017	SE, MET	*HvDEP2-4*
	***qGw2-3***	2H	647.83–653.98	42.20–203.95	43.11–84.33	+	2016, 2017, 2018	SE, MET, Ct	*Vrs1/Int-d*
	***qGw3-1***	3H	682.23–683.15	3.17–6.53	0.58–2.50	+	2016, 2018	SE, MET, Ct	*Hvemp5; HvTGW6-3*
	*qGw3-3*	3H	660.74–664.33	4.54–12.98	1.40–2.56	+	2016, 2017	SE, MET, Rt, Rt+Ct	*HvOsBDG1*
	*qGw3-4*	3H	631.86–641.54	5.02–8.00	0.70–3.01	+	2016, 2018	SE, MET, Ct, Rt+Ct	*sdw1/denso; Hvvp1*
	*qGw4-1*	4H	529.57–532.00	3.01–14.23	1.08–3.04	−	2017, 2018	SE, MET, Rt	
	*qGw6-1*	6H	565.70–566.84	3.52–6.17	0.51–1.67	−	2016, 2017	SE, MET	*HvOsbHLH107; HvBLS1*
	*qGw7-1*	7H	546.66–552.07	17.71–49.55	6.73–10.86	+	2016, 2017, 2018	SE, MET, Rt	*Nud; HvTaMOC1*
Lwr	*qLwr1-1*	1H	415.73–417.54	3.79–18.14	3.20–9.67	+	2016, 2017, 2018	SE, MET, Rt, Ct, Rt+Ct	*HvCO9; HvSMOS1*
	***qLwr2-1***	2H	647.83–653.98	16.01–89.73	17.11–50.47	−	2016, 2017, 2018	SE, MET, Ct	*Vrs1/Int-d*
	*qcLwr2-2*	2H	659.60–659.72	3.06–3.91	1.76–2.71	+	2016, 2017, 2018	Rt, Rt+Ct	*HvGS2-3*
	*qLwr3-1*	3H	666.33–670.02	3.74–5.49	1.31–2.84	−	2017, 2018	SE, MET, Rt, Rt+Ct	*HvOsBDG1*
	*qLwr3-2*	3H	594.52–603.14	3.14–11.29	1.90–4.43	+	2016, 2018	SE, MET, Rt+Ct	
	***qLwr7-2***	7H	511.81–514.43	9.86–56.05	12.55–43.65	+	2016, 2018	SE, MET, Rt	
	***qcLwr7-5***	7H	345.67–381.62	9.49–11.05	19.19–24.55	+	2016, 2018	Ct, Rt+Ct	*btwd1*
Ga	*qGa1-2*	1H	18.12–18.79	3.04–6.39	0.71–1.70	−	2017, 2018	SE, MET, Rt, Rt+Ct	
	***qGa2-1***	2H	647.83–653.98	32.16–119.3	30.04–56.57	+	2016, 2017, 2018	SE, MET, Ct	*Vrs1/Int-d*
	*qGa3-1*	3H	660.74–664.33	3.11–12.05	1.02–2.66	+	2016, 2017	SE, MET, Rt, Rt+Ct	*HvOsBDG1*
	*qGa3-2*	3H	651.82–658.11	3.24–15.75	0.57–2.46	+	2017, 2018	SE, MET, Rt, Ct, Rt+Ct	
	*qGa3-3*	3H	585.40–590.90	3.55–10.6	0.85–1.58	+	2017, 2018	SE, MET, Ct	
	*qcGa3-11*	3H	285.88–303.30	3.48–6.39	1.00–1.83	−	2017, 2018	Rt, Ct, Rt+Ct	
	*qGa4-1*	4H	529.57–532.00	3.21–24.91	0.59–5.91	−	2017, 2018	SE, MET, Rt, Ct, Rt+Ct	
	***qGa7-1***	7H	546.66–559.76	16.04–85.88	16.45–34.52	+	2016, 2017, 2018	SE, MET, Rt	*Nud; HvTaMOC1*
	***qGa7-2***	7H	345.67–381.62	5.28–89.98	3.19–29.04	+	2016, 2017, 2018	SE, MET, Rt, Ct	*btwd1*
Gp	***qGp2-1***	2H	647.83–653.98	12.96–62.67	9.6–25.83	+	2016, 2017, 2018	SE, MET, Ct	*Vrs1/Int-d*
	*qGp3-2*	3H	624.57–624.79	3.23–7.52	1.08–1.99	+	2016, 2017	SE, MET, Rt	*Vrn-H3/Sgh3*
	*qcGp3-8*	3H	285.88–303.30	5.60–11.58	0.72–13.95	−	2017, 2018	Rt, Ct, Rt+Ct	
	***qGp7-1***	7H	546.66–563.86	16.42–138.45	16.85–40.26	+	2016, 2017, 2018	SE, MET, Rt	*Nud; HvTaMOC1*
	***qGp7-2***	7H	461.41–471.57	11.37–51.06	3.29–26.18	+	2017, 2018	SE, MET	
	*qGp7-6*	7H	166.04–169.40	7.37–37.22	3.70–18.72	+	2016, 2017	SE, MET, Rt, Rt+Ct	
Gd	***qGd2-1***	2H	647.83–653.98	26.62–110.94	22.56–72.22	+	2016, 2017, 2018	SE, MET, Ct	*Vrs1/Int-d*
	*qGd3-2*	3H	651.82–654.70	4.01–10.67	1.03–2.78	+	2016, 2017, 2018	SE, MET, Rt, Ct, Rt+Ct	
	*qGd3-3*	3H	631.86–640.23	4.61–7.74	0.59–2.83	+	2016, 2018	SE, MET	*sdw1/denso; Hvvp1*
	*qGd4-1*	4H	529.57–532.00	5.87–6.52	1.19–1.33	−	2017, 2018	SE, MET, Rt+Ct	
	***qGd7-1***	7H	546.66–562.79	39.99–61.16	25.24–30.26	+	2016, 2017, 2018	SE, MET	*Nud; HvTaMOC1*
	***qGd7-5***	7H	345.67–381.62	10.24–66.39	4.92–43.79	+	2016, 2017, 2018	SE, MET, Rt, Ct	*btwd1*
Gr	*qGr1-1*	1H	415.73–417.54	6.48–16.76	6.14–8.57	−	2016, 2018	SE, MET	*HvCO9; HvSMOS1*
	***qGr2-1***	2H	647.83–653.98	13.95–74.96	18.3–56.05	+	2016, 2017, 2018	SE, MET, Ct	*Vrs1/Int-d*
	***qGr7-2***	7H	511.81–514.43	21.91–31.74	13.01–36.2	−	2016, 2018	SE, MET	
	***qGr7-5***	7H	345.67–381.62	24.76–58.94	15.55–46.83	+	2017, 2018	SE, MET	*btwd1*
Ffd	*qFfd1-2*	1H	320.30–320.80	4.34–9.87	3.24–3.42	−	2016, 2017	SE, MET	
	***qFfd2-1***	2H	647.83–653.98	27.21–121.71	52.70–73.72	+	2016, 2017, 2018	SE, MET, Ct	*Vrs1/Int-d*
	*qFfd3-4*	3H	91.96–105.75	3.45–11.95	2.50–8.57	−	2016, 2017	SE, MET, Rt+Ct	
	***qFfd7-1***	7H	511.81–514.43	18.01–18.11	4.40–23.29	−	2016, 2018	SE, MET, Rt	

*^a^QTLs in bold indicate the major QTL with explaining more than 10% of the phenotype variation (mean value from all QTLs). ^b^Chromosome. ^c^The phenotypic variation explained (in %) by each QTL. ^d^Additive effect; positive values indicate that the alleles coming from Huadamai 6; negative values indicated that the alleles coming from Huaai 11. ^e^SE, single-environment QTL analysis; MET, multi-environment trials (MET) analysis; Rt, covariate QTL analysis using row type as a covariate; Ct, covariate QTL analysis using caryopsis type as a covariate; Rt+Ct, covariate QTL analysis using row type and caryopsis type as a covariate.*

### QTL Hotspots of Barley Grain Size and Weight Trait

In this study, we found a considerable number of overlapping QTLs for different traits. By integrating all the QTLs identified using the five mapping methods, we found 27 hotspots on six chromosomes involving 421 QTLs, including 3, 2, 10, 1, 2, and 9 hotspots for 1H, 2H, 3H, 4H, 5H, and 7H, respectively (Table 4). For instance, the significant QTL hotspots on chromosome 2H at 124.5–128.5 cM (647.83–653.98 Mb) and on chromosome 7H at 64.5–69.5 cM (546.66–563.86 Mb), included 61 QTLs and 35 QTLs, each influenced all nine grain size and weight traits simultaneously. Another significant QTL hotspot including 28 QTLs, detected on chromosome 7H at 150.5–153.5 cM (345.67–381.62 Mb), was related to Tgw, Gl, Lwr, Ga, Gp, Gd, and Gr. Similarly, the rest of QTL hotspots each affected more than three or more grain traits.

**Table 4:** QTL hotspots for grain size and weight traits identified in the barley genome.

Hotspot^a^	Chr.	Genetic interval (cM)	Physical interval (Mb)	No.^b^	Involved traits	Mapping method	Candidate genes or orthologs	Previous QTL or Hotspot ^c^
1_1	1H	22.5–23.5	415.25–423.42	20	Tgw, Gw, Lwr, Ga, Gd, Gr, Ffd	SE, MET, Rt, Ct, Rt+Ct	*HvCO9; HvSMOS1*	
1_2	1H	126.5–128.5	21.61–22.26	6	Tgw, Gw, Ga	SE, MET, Rt	*HvDEP2-4*	
1_3	1H	129.5–133.5	18.12–18.79	22	Tgw, Gl, Gw, Ga, Gp, Gd	SE, MET, Rt, Ct, Rt+Ct		
2_1	2H	124.5–128.5	647.83–653.98	61	Tgw, Gl, Gw, Lwr, Ga, Gp, Gd, Gr, Ffd	SE, MET, Ct	*Vrs1/Int-d*	*qKA-2H, qKW-2H, qKL-2H, qKP-2H, qKFS-2H, qKFC-2H, qTKW-2H*, (Ayoub et al., 2002); *qKWT.ak-2H, qKLN.ak-2H, qKWD.ak-2H, qKA.ak-2H, qKS.ak-2H*, (Sameri and Komatsuda, 2007); *Qtkw-2H* (Comadran et al., 2011); *qTgw2-1, qTgw2-2* (Wang S. et al., 2016); *2_3* (Xu et al., 2018); *qtnTGW-2H-20* (Hu et al., 2018)
2_2	2H	159.5–160.5	695.60–695.72	7	Gl, Lwr, Gp	Rt, Ct, Rt+Ct	*HvGS2-3*	
3_1	3H	17.5–21.5	661.19–670.02	28	Tgw, Gl, Gw, Ga, Gp, Gd	SE, MET, Rt, Ct, Rt+Ct	*HvOsBDG1*	
3_2	3H	23.5–27.5	651.82–658.11	20	Tgw, Gl, Ga, Gp, Gd	SE, MET, Rt, Ct, Rt+Ct		
3_3	3H	29.5–33.5	631.86–646.92	19	Tgw, Gl, Gw, Ga, Gp, Gd, Ffd	SE, MET, Rt, Ct, Rt+Ct	*sdw1/denso; Hvvp1*	*LEN-3H* (Zhou et al., 2016); *QTGW. MC3H.1* (Mikolajczak et al., 2016); *QTL-3H-9* (Maurer et al., 2016)
3_4	3H	36.5–38.5	623.15–624.57	6	Tgw, Gl, Ga, Gp	SE, MET, Rt, Ct	*Vrn-H3/Sgh3*	*qGA-3H* (Xu et al., 2018)
3_5	3H	47.5–49.5	604.49–606.01	12	Gl, Lwr, Gp	SE, MET, Rt, Ct, Rt+Ct	*HvAUX1*	
3_6	3H	52.5–58.5	585.40–603.14	18	Gl, Lwr, Ga, Gp, Gr	SE, MET, Rt, Ct, Rt+Ct		
3_7	3H	83.5–84.5	511.22–512.91	10	Gl, Gw, Ga, Gp, Gd	SE, MET, Rt+Ct	*HvD61-1; HvD61-2; HvD61-3*	*qGL-3H, qGR-3H, qGY-3H* (Xu et al., 2018)
3_8	3H	110.5–113.5	339.95–341.50	8	Gl, Gw, Ga, Gp, Gd	SE, MET, Rt, Ct	*HvRGB1-2*	
3_9	3H	155.5–157.5	285.88–303.30	16	Tgw, Gl, Gw, Ga, Gp	Rt, Ct, Rt+Ct		
3_10	3H	190.5–193.5	91.96–105.75	10	Tgw, Gw, Ga, Ffd	SE, MET, Rt, Rt+Ct		
4_1	4H	58.5–62.5	529.57–532.00	21	Tgw, Gw, Ga, Gd	SE, MET, Rt, Ct, Rt+Ct		
5_1	5H	0.0–0.5	0.43–2.57	10	Tgw, Ga, Gd	SE, MET, Rt, Ct, Rt+Ct		
5_2	5H	202.5–203.5	533.43–535.45	5	Gl, Gp, Gr	SE, MET, Ct, Rt+Ct	*Hvemp6*	*QTL-GT1, QTL-P1* (Watt et al., 2018)
7_1	7H	64.5–69.5	546.66–563.86	35	Tgw, Gl, Gw, Lwr, Ga, Gp, Gd, Gr, Ffd	SE, MET, Rt	*Nud; HvTaMOC1*	*qTgw7-1* (Wang S. et al., 2016)
7_2	7H	79.5–80.5	511.81–514.43	12	Gl, Lwr, Gp, Gr, Ffd	SE, MET, Rt		
7_3	7H	93.5–96.5	461.41–471.57	5	Tgw, Gp, Gd	SE, MET, Rt		
7_4	7H	133.5–136.5	382.25–410.75	12	Gl, Lwr, Gp	SE, MET, Ct, Rt+Ct		
7_5	7H	150.5–153.5	345.67–381.62	28	Tgw, Gl, Lwr, Ga, Gp, Gd, Gr	SE, MET, Rt, Ct, Rt+Ct	*btwd1*	
7_6	7H	163.5–166.5	166.04–169.40	19	Tgw, Gl, Lwr, Gp, Gr	SE, MET, Rt, Rt+Ct		
7_7	7H	178.5–179.5	103.21–109.54	5	Gl, Ga, Gp	Ct, Rt+Ct		
7_8	7H	181.5–185.5	81.34–87.38	9	Tgw, Ga, Ffd	SE, MET, Ct, Rt+Ct		*qTgw7-3, qTgw7-4 (*Wang S. et al., 2016*)*
7_9	7H	224.5–226.5	4.97–7.56	5	Tgw, Ga, Gd	SE, MET, Ct, Rt+Ct	*HvGS3; HvTaGS-D1*	*7_1* (Xu et al., 2018)

*^a^The first figure indicated the chromosome where the QTL hotspot is located, and the second indicated the order of the QTL hotspots in the corresponding chromosome. ^b^The number of QTLs included in each hotspot. ^c^Some studies have not named QTL, we renamed it in the format “q+ trait + chromosome.”*

### Genetic Correspondence in Diverse Cereals

**FIGURE 4:**
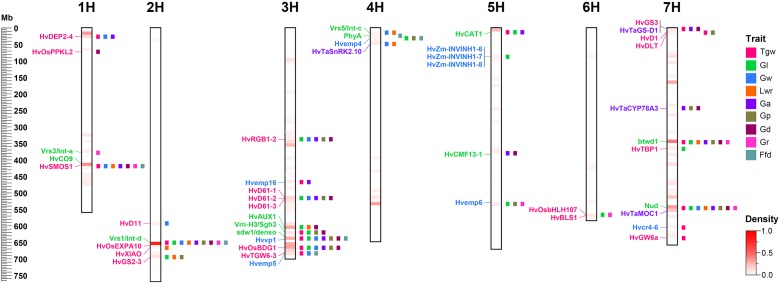
Comparative analysis of QTLs detected in this study with barley yield-associated genes and barley orthologs of grain size or weight genes from rice, wheat, and maize. A total of 20 rice orthologs (shown in red), 4 wheat orthologs (shown in blueviolet), 9 maize orthologs (shown in dodgerblue), and 12 barley genes (shown in lime) were shown in the whole barley genome. The heat map in the chromosome region illustrated the density of QTLs for nine grain size and weight traits. The window size was 10 Mb.

To identify candidate genes for QTLs and their correspondence to grain size/weight related genes in other cereal species, we collected 38 barley genes and 148 other cereal crops genes (including 94 rice genes, 40 maize genes, and 14 wheat genes) associated with grain size/weight or yield (Supplementary Tables S7, S8). Based on the Ensembl Plant Database (see footnote 1), we identified 164 barley orthologs from 112 other cereal species genes, including 102, 48, and 14 barley orthologs for 70 rice genes, 29 maize genes, and 13 wheat genes, respectively, while no corresponding ortholog was found for 36 genes (Supplementary Table S8 and Supplementary Figure S2). Out of these 112 genes, 93, 7, 5, 2, 1, 1, 2, and 1 gene have one, two, three, four, five, six, seven, and nine orthologs, respectively. We mapped these 38 barley genes and 164 barley orthologs to the barley physical map and aligned these against our QTLs, and identified 45 barley genes or orthologs that were located within ±5 Mb on either side of the QTL peak, including 12, 20, 9, and 4 genes or orthologs for barley, rice, maize, and wheat, respectively (Table 5 and Figure 4). Importantly, 20 of them were located in the 14 QTL hotspot regions on chromosome 1H, 2H, 3H, 5H, and 7H.

**Table 5:** Correspondence between QTL of barley grain size and weight and known genes of other cereal grain traits.

No.	Barley genes or orthologs	Barley gene_ID	Chr.	Position (Mb)	Regulated traits in this study	QTL hotspot	Gene name	Species	Gene_ID	Regulated traits ^a^	Functional annotation
1	*HvDEP2-4*	HORVU1Hr1G011030	1H	26.22	Tgw, Gw, Ga	1_2	*DEP2/EP2/SRS1*	Rice	Os07g0616000	Small and round grain	Protein Of 1365 Amino Acids With Unknown Function
2	*HvOsPPKL2*	HORVU1Hr1G019330	1H	72.53	Gd		*OsPPKL2*	Rice	Os05g0144400	Gl	Serine/Threonine-Specific Protein Phosphatase And Bis(5-Nucleosyl)-Tetraphosphatase Domain Containing Protein
3	*Vrs3/Int-a*	MLOC_69611	1H	378.44	Gr		*Vrs3/Int-a*	Barley	MLOC_69611	Lateral spikelet fertility	Lysine-Specific Demethylase 5B
4	*HvCO9*	HORVU1Hr1G056120	1H	411.26	Tgw, Gw, Lwr, Ga, Gd, Gr, Ffd	1_1	*HvCO9*	Barley	HORVU1Hr1G056120	Flowering time	Zinc Finger Protein Constans-Like 4
5	*HvSMOS1*	HORVU1Hr1G056500	1H	414.51	Tgw, Gw, Lwr, Ga, Gd, Gr, Ffd	1_1	*SMOS1/SHB/RLA1*	Rice	Os05g0389000	Small grain	Ap2/Erf Transcription Factor
6	*HvD11*	HORVU2Hr1G081650	2H	592.33	Gw		*D11/CPB1/CYP724B1/GNS4*	Rice	Os04g0469800	Gl	Cytochrome P450 (Cyp724B1) Enzyme; Brassinosteroid (Br) Biosynthesis
7	*Vrs1/Int-d*	HORVU2Hr1G092290	2H	652.03	Tgw, Gl, Gw, LWr, Ga, Gp, Gd, Gr, Ffd	2_1	*Vrs1/Int-d*	Barley	HORVU2Hr1G092290	Lateral spikelet fertility	Homeobox-Leucine Zipper Protein Family
8	*HvOsEXPA10*	HORVU2Hr1G093690	2H	659.20	Lwr		*OsEXPA10*	Rice	Os04g0583500	Gl, Tgw	Al-Inducible Expansin
9	*HvXIAO*	HORVU2Hr1G094360	2H	664.45	Lwr		*XIAO*	Rice	Os04g0576900	Small grain	Lrr Kinase
10	*HvGS2-3*	HORVU2Hr1G101770	2H	694.69	Gl, Lwr, Gp	2_2	*GS2/OsGRF4/GL2/GLW2*	Rice	Os02g0701300	Gl, Gw	Rice Growth Regulatin G Factor 4 (Osgrf 4)
11	*HvRGB1-2*	HORVU3Hr1G048710	3H	337.04	Gl, Gw, Ga, Gp, Gd	3_8	*RGB1*	Rice	Os03g0669100	Small grain	G Protein B Subunit
12	*Hvemp16*	HORVU3Hr1G061620	3H	468.80	Tgw, Ga		*emp16/ZmPPR445*	Maize	Zm00001d011559	Empty pericarp	Uncharacterized Protein
13	*HvD61-1*	HORVU3Hr1G068000	3H	516.13	Gl, Gw, Ga, Gp, Gd	3_7	*D61/OsBRI1*	Rice	Os01g0718300	Small grain	Brassinosteroid Lrr Receptor Kinase
14	*HvD61-2*	HORVU3Hr1G068010	3H	516.21	Gl, Gw, Ga, Gp, Gd	3_7	*D61/OsBRI1*	Rice	Os01g0718300	Small grain	Brassinosteroid Lrr Receptor Kinase
15	*HvD61-3*	HORVU3Hr1G068020	3H	516.35	Gl, Gw, Ga, Gp, Gd	3_7	*D61/OsBRI1*	Rice	Os01g0718300	Small grain	Brassinosteroid Lrr Receptor Kinase
16	*HvAUX1*	HORVU3Hr1G084750	3H	608.94	Gl, Lwr, Gp	3_5	*HvAUX1*	Barley	HORVU3Hr1G084750	Gf	Putative auxin permease-like t
17	*Vrn-H3/Sgh3*	HORVU3Hr1G087100	3H	619.90	Tgw, Gl, Ga, Gp	3_4	*Vrn-H3/Sgh3*	Barley	HORVU3Hr1G087100	Grain yield	Flowering Locus T1
18	*sdw1/denso*	HORVU3Hr1G090980	3H	634.08	Tgw, Gl. Gw, Ga, Gp, Gd, Ffd	3_3	*sdw1/denso*	Barley	HORVU3Hr1G090980	Grain yield	Gibberellin 20-Oxidase 3
19	*Hvvp1*	HORVU3Hr1G092690	3H	639.79	Tgw, Gl. Gw, Gp, Gd, Ffd	3_3	*vp1/vp4/ZmABI1*	Maize	Zm00001d042396	Kernel development	Regulatory Protein Viviparous-1
20	*HvOsBDG1*	HORVU3Hr1G104350	3H	666.35	Tgw, Gl, Gw, Ga, Gp, Gd	3_1	*OsBDG1*	Rice	Os11g0514400	Small grain	Similar To Brassinosteroid Insensitive 1-Associated Receptor Kinase 1
21	*HvTGW6-3*	HORVU3Hr1G109810	3H	679.40	Tgw, Gw, Ffd		*TGW6*	Rice	Os06g0623700	Gl, Gf, Tgw	Protein With Indole-3-Acetic Acid (Iaa)-Glucose Hydrolase Activity
22	*Hvemp5*	HORVU3Hr1G110410	3H	681.60	Tgw, Gw		*emp5*	Maize	Zm00001d042039	Empty pericarp	Emp5 Protein
23	*Vrs5/Int-c*	HORVU4Hr1G007040	4H	17.60	Gw, Lwr		*Vrs5/Int-c*	Barley	HORVU4Hr1G007040	Lateral spikelet fertility	Transcription Factor Teosinte Branched 1
24	*PhyA*	HORVU4Hr1G008610	4H	23.60	Ffd		*PhyA*	Barley	HORVU4Hr1G008610	Grain yield	Phytochrome A
25	*Hvemp4*	HORVU4Hr1G009900	4H	29.01	Gl. Gp, Ffd		*emp4/ZmPPR069*	Maize	Zm00001d033869	Empty pericarp	Pentatricopeptide Repeat-Containing Protein
26	*HvTaSnRK2.10*	HORVU4Hr1G013540	4H	47.80	Gw, Lwr		*TaSnRK2.10*	Wheat	TraesCS4A02G235600; TraesCS4B02G079300; TraesCS4D02G078100	Tgw	SNF1-type serine-threonine protein kinase
27	*HvCAT1*	HORVU5Hr1G006900	5H	12.45	Tgw, Gw, Ga		*HvCAT1*	Barley	HORVU5Hr1G006900	Gf	Cationic Amino Acid Transporter 2
28	*HvZm-INVINH1-6*	HORVU5Hr1G019700	5H	87.07	Gl		*Zm-INVINH1*	Maize	Zm00001d002517	Kernel development	Cell Wall/Vacuolar Inhibitor Of Fructosidase 2
29	*HvZm-INVINH1-7*	HORVU5Hr1G019720	5H	87.08	Gl		*Zm-INVINH1*	Maize	Zm00001d002517	Kernel development	Cell Wall/Vacuolar Inhibitor Of Fructosidase 2
30	*HvZm-INVINH1-8*	HORVU5Hr1G019750	5H	87.14	Gl		*Zm-INVINH1*	Maize	Zm00001d002517	Kernel development	Cell Wall/Vacuolar Inhibitor Of Fructosidase 2
31	*HvCMF13-1*	HORVU5Hr1G049330	5H	382.76	Ga, Gd		*HvCMF13-1*	Barley	HORVU5Hr1G049330	Flowering time	CCT motif -containing response regulator protein
32	*Hvemp6*	HORVU5Hr1G070460	5H	528.93	Gl, Gp, Gr	5_2	*emp6*	Maize	Zm00001d005959	Empty pericarp	Empty pericarp 6
33	*HvOsbHLH107*	HORVU6Hr1G088020	6H	565.62	Gw, Gr		*OsbHLH107*	Rice	Os02g0805250	Gl, Tgw	Nucleus-Localized Bhlh Transcription Factor
34	*HvBLS1*	HORVU6Hr1G088790	6H	567.41	Gw, Gr		*BLS1/BSG1/OsG1L6/TH1/AFD1*	Rice	Os02g0811000	Gw, Gt, Tgw	Duf640 Domain Containing Protein; Alog Domain-Containing Nuclear Protein
35	*HvGS3*	HORVU7Hr1G001910	7H	3.94	Tgw, Ga, Gd	7_9	*GS3*	Rice	Os03g0407400	Gl, Gw, Gt, Tgw	Protein with plant-specific organ size regulation (OSR) domain, transmembrane region, TNFR/NGFR family cysteine-rich domain and VWFC module
36	*HvTaGS-D1*	HORVU7Hr1G001910	7H	3.94	Tgw, Ga, Gd	7_9	*TaGS-D1*	Wheat	TraesCS7D02G015000	Gl, Tgw	Putative Transmembrane Protein With A Pebp-Like Domain
37	*HvD1*	HORVU7Hr1G008720	7H	11.33	Tgw, Gp		*D1/RGA1*	Rice	Os05g0333200	Small grain	G Protein A Subunit
38	*HvDLT*	HORVU7Hr1G010620	7H	14.94	Tgw, Gp		*DLT/OsGRAS-32/D62/ GS6/SMOS2*	Rice	Os06g0127800	Gw, Tgw	Gai-Rga-Scr (Gras) Family Protein; Brassinosteroid Signaling
39	*HvTaCYP78A3*	HORVU7Hr1G057100	7H	243.84	Gp, Gd, Gr		*TaCYP78A3*	Wheat	TraesCS7A02G270700; TraesCS7B02G168800; TraesCS7D02G271100	Gl, Gw	Cytochrome P450 (Cyp78A3) Enzym
40	*btwd1*	−	7H	345.67	Tgw, Gl, Lwr, Ga, Gp, Gd, Gr	7_5	*btwd1*	Barley	N/A	Grain yield	
41	*HvTBP1*	HORVU7Hr1G068990	7H	366.10	Gl		*OsBISERK1/OsSERK1/OsBAK1/TBP1*	Rice	Os08g0174700	Gl, Gw, Gn	A Member Of The Somatic Embryogenesis Receptor Kinases (Serks) Family; Brassinosteroid (Br) Signaling
42	*Nud*	HORVU7Hr1G089930	7H	546.59	Tgw, Gl, Gw, Lwr, Ga, Gp, Gd, Gr, Ffd	7_1	*Nud*	Barley	HORVU7Hr1G089930	Covered/naked caryopsis	Ethylene-responsive transcription factor 1
43	*HvTaMOC1*	HORVU7Hr1G091000	7H	555.11	Tgw, Gl, Gw, Lwr, Ga, Gp, Gd, Gr, Ffd	7_1	*TaMOC1*	Wheat	TraesCS7B02G285500	Spikelet number per spike	Typical Nucleus Localized Protein
44	*Hvcr4-6*	HORVU5Hr1G097470	7H	604.44	Tgw		*cr4*	Maize	Zm00001d023425	Opaque endosperm	Putative Receptor Protein Kinase CRINKLY4
45	*HvGW6a*	HORVU7Hr1G113480	7H	637.08	Tgw		OsglHAT1/GW6a	Rice	Os06g0650300	Tgw	Histone H4 Acetyltransferase

*^a^Gf, grain filling; Gt, grain thickness; Gn, grain number.*

## Discussion

Improving barley yield has always been an important objective of barley genetic research and breeding programs. Grain size is one of the key factors determining barley yield. Although some QTLs related to grain size and weight have been identified, and several genes affecting grain size or weight have been characterized, our knowledge on the genetic and molecular mechanisms that regulate grain size in barley remain largely unknown. In this study, we have identified 593 QTLs for nine barley grain size and weight traits using five mapping methods. A total of 45 potential candidate genes were identified, providing important insight into the genetic basis of barley grain size and weight.

### QTLs for Grain Size and Weight

QTL analysis of thousand grain weight (Tgw) has been performed previously (Ayoub et al., 2002; Coventry et al., 2003; Chen et al., 2004; Li et al., 2005, 2006; Sameri and Komatsuda, 2007; Comadran et al., 2011; Kalladan et al., 2013; Walker et al., 2013; Maurer et al., 2016; Mikolajczak et al., 2016; Wang J. et al., 2016; Xu et al., 2018), but some important information is still missed for the QTL of grain size in barley. To date, QTLs for grain length (Gl) (Ayoub et al., 2002; Sameri and Komatsuda, 2007; Kalladan et al., 2013; Walker et al., 2013; Zhou et al., 2016; Watt et al., 2018; Xu et al., 2018), grain width (Gw) (Ayoub et al., 2002; Sameri and Komatsuda, 2007; Kalladan et al., 2013; Walker et al., 2013; Cu et al., 2016; Xu et al., 2018), grain length-width ratio (Lwr) (Sameri and Komatsuda, 2007; Kalladan et al., 2013), grain area (Ga) (Ayoub et al., 2002; Sameri and Komatsuda, 2007; Xu et al., 2018), grain diameter (Gd) (Cu et al., 2016), and grain roundness (Gr) (Ayoub et al., 2002) have been mapped on almost all seven linkage groups, while QTLs conferring grain perimeter (Gp) and factor form density (Ffd) were rarely reported previously in barley.

In the present study, we successfully identified 60 reliable QTLs for the nine traits of grain size and weight and found 27 hotspot regions that distributed on chromosome 1H, 2H, 3H, 4H, 5H, and 7H (Tables 3, 4). Comparing our QTLs with published results by using BARLEYMAP pipeline (Cantalapiedra et al., 2015), we found some QTLs identified here appeared to coincide to the QTLs described previously. For example, 61 QTLs encompassing all nine traits were clustered in the interval of 647.83–653.98 Mb on chromosome 2H, which corresponds to the QTLs reported previously (Ayoub et al., 2002; Sameri and Komatsuda, 2007; Comadran et al., 2011; Wang J. et al., 2016; Xu et al., 2018), indicating its stability and major effects. By using genome-wide association analysis, Xu et al. (2018) detected a QTL hotspot 7_1 for grain size and weight traits near the barley ortholog of rice *Gs3* gene on chromosome 7H (HORVU7Hr1G001910), which may be the same to the QTL hotspot 7_9 (7H: 4.97–7.56 Mb) identified in our study. The QTL hotspot 3_3 consisting of 19 QTLs for Tgw, Gl, Gw, Ga, Gp, Gd, and Ffd on chromosome 3H at 29.5–33.5 cM (631.86–646.92 Mb) identified here may share the same location with the QTL *LEN-3H* for Gl described by Zhou et al. (2016), the QTL *QTGW. MC3H.1* for Tgw detected by Mikolajczak et al. (2016), and the QTL *QTL-3H-9* for Tgw found by Maurer et al. (2016).

In addition, some novel QTLs were also identified in our study. For example, QTL hotspot 4_1 for Tgw, Gw, Ga, and Gd on chromosome 4H at 529.57–532.00 Mb identified here are different from the QTLs or QTL hotspots for grain size and weight described on chromosome 4H in previous reports (Maurer et al., 2016; Mikolajczak et al., 2016; Zhou et al., 2016; Xu et al., 2018). Similarly, QTL hotspot 7_2 (511.81–514.43 Mb) and 7_5 (345.67–381.62 Mb) on chromosome 7H contained 12 QTLs and 28 QTLs, respectively, which are different from those QTLs for grain size and weight detected on chromosome 7H in previous studies (Mikolajczak et al., 2016; Zhou et al., 2016; Xu et al., 2018).

### Some Barley Yield-Related Genes Were Associated With Grain Size and Weight

Many genes have been proved to have pleiotropic effects. For example, semi-dwarf gene *sdw1/denso* controls plant height, the number of tillers, grain yield, and grain size (Forster, 2001; Kuczyńska et al., 2013; Kuczyńska et al., 2014). Photoperiod response gene *Ppd-H1/Eam1/HvPRR37* has pleiotropic effects on flowering time, leaf size, and yield components (Li et al., 2005; Digel et al., 2016). Thus, we investigated 38 barley genes, including 37 genes that were previously described to influence grain yield and a novel dwarf gene *btwd1* (Ren et al., 2016) previously identified in our DH population, to explore whether they also affect barley grain size and weight (Supplementary Table S7). Among these 38 barley genes, 12 were identified as potential candidate genes affecting barley grain size and weight (Table 5). Importantly, seven of them were identified in the QTL hotspot region controlling barley grain size and weight.

The most important QTL hotspot 2_1 on chromosome 2H at 124.5–128.5 cM (647.83–653.98 Mb) contained 61 QTLs for all grain size and weight traits. The *vrs1/int-d* gene (HORVU2Hr1G092290) was also mapped to this region, which has previously been reported to affect row type, grain size and Tgw (Ayoub et al., 2002; Komatsuda et al., 2007; Sakuma et al., 2017; Hu et al., 2018; Xu et al., 2018). Another significant QTL hotspot region underlying all grain size and weight traits on chromosome 7H at 64.5–69.5 cM (546.66–563.86 Mb) was physically close to *nud* gene (HORVU7Hr1G089930) that determines the hulled/naked caryopsis phenotype (Taketa et al., 2008). Several studies have previously reported that yield-related QTLs, including Tgw, were tightly linked to *nud* gene (Barabaschi et al., 2012; Gong et al., 2016). Since our population was derived from a cross between naked six-rowed barley and hulled two-rowed barley, the effects of these two genes on grain traits were in line with our expectations. Due to large effect of these two genes in the population used here, the effect of other QTLs on grain traits was relatively weak and difficult to detect. Therefore, to reduce the confounding effects of these two genes on grain traits, we carried out covariate QTL analysis to find more QTLs association with grain traits. In fact, our strategy was successful because we found more than 60% (189 QTLs) of the covariate QTLs were new QTLs that were not detected in either single-environment QTL analysis or MET analysis (Supplementary Table S6). Although these QTLs had relatively weak effects on the grain traits in this population due to the large influence of the *vrs1/int-d* and *nud* genes, they played an important role in revealing the genetic basis of barley grain traits. For mapping barley grain traits, it is better to use parents with the same row type and caryopsis type to construct mapping population to eliminate the influence of these two genes, *vrs1/int-d* and *nud*, over other QTLs.

Except for *vrs1/int-d* and *nud* genes, the vernalization gene *Vrn-H3*/*Sgh3/HvFT1* (HORVU3Hr1G087100) and flowering gene *HvCO9*/*HvCMF11* (HORVU1Hr1G056120), which are associated with barley yield, were physically close to QTL hotspot regions 1_1 (415.25–423.42 Mb) and 3_4 (623.15–624.57 Mb), respectively (Table 5). Moreover, we also found that semi-dwarf gene *sdw1/denso* (HORVU3Hr1G090980) and dwarf gene *btwd1* [close to SNP marker 7HL_6335336 (7H: 345673515–345673602 bp)] were located in the QTL hotspots region of 3_3 (631.86–646.92 Mb) and 7_5 (345.67–381.62 Mb), respectively (Table 5). These two dwarf or semi-dwarf genes have previously been described to be closely related to yield, and the *sdw1/denso* semi-dwarf gene has also been reported to have pleiotropic or tightly linkage effects on Tgw and grain size (Forster, 2001; Coventry et al., 2003; Kuczyńska et al., 2013; Maurer et al., 2016; Ren et al., 2016). We predicted 20 candidate genes for *btwd1* that were located within ±5 Mb on either side of the nearest SNP marker 7HL_6335336 of the *btwd1* gene (Supplementary Table S9). Among them, the most promising candidate gene HORVU7Hr1G066930 (7H: 346181113–346195444 bp) whose annotation information is WD-40 repeat protein-like isoform 1, is an ortholog of rice gene *OsTPL/ASP1/OsLIS-L1*. The rice *OsTPL/ASP1/OsLIS-L1* gene encoding a lissencephaly type-1-like protein and containing the WD40 motif has previously been confirmed to regulate the first internode elongation of rice, resulting in a dwarf phenotype (Gao et al., 2012). These results provided evidence that many of the yield-related genes might have pleiotropic or tightly linkage effects on barley grain size and weight and contributed to phenotypic diversity in barley grain size and weight.

### Some Grain Size/Weight Related Genes in Other Cereals May Have Conserved Functions in Barley

Comparative genomics has demonstrated that orthologs from common ancestors generally have conserved functions, which provides an effective strategy for the discovery of barley genes (Devos, 2005; Su et al., 2011; Murat et al., 2017). For example, the rice gene *DEP1* that encodes a highly cysteine-rich G protein gamma subunit to regulate grain yield (Kunihiro et al., 2013), its orthologs gene *HvDep1* in barley has also been found to have similar functions as in rice (Bélanger et al., 2014; Wendt et al., 2016). At present, a considerable number of genes associated with grain size/weight have been characterized in rice, maize, and wheat. Thus, identification of orthologs for these genes in the barley genome, using comparative genomic approaches, may provide more insights into the genetic mechanisms of barley grain size and weight.

In this study, a total of 32 barley orthologs were identified as potential candidate genes that determine barley grain size or weight, including 20 (one of them is the same as in wheat), 9 and 4 genes from rice, maize, and wheat, respectively (Table 5). The barley ortholog of the rice *OsBDG1* gene is on chromosome 3H at 666.35 Mb (HORVU3Hr1G104350), which encodes the leucine-rich repeat receptor-like protein kinase family protein and is nearby the QTL hotspot 3_1 (661.19–670.02 Mb) identified in this study (Table 5). The rice *OsBDG1* gene encoding a small protein with short leucine-rich-repeats possessing cell elongation activity, has previously been proven to positively regulate grain size in rice (Jang and Li, 2017). Hence, we believe that HORVU3Hr1G104350 should be a reliable candidate gene affecting grain size as the function of the *OsBDG1* gene. The barley ortholog of the rice *GS3* gene, HORVU7Hr1G001910, located on chromosome 7H at 3.94 Mb, is close to the QTL hotspot 7_9 (7H: 4.97–7.56 Mb) for Tgw, Ga, and Gd, encoding a grain length protein (Table 5). The rice *GS3* gene encoding a membrane protein with several conserved domains including the plant-specific organ size regulation (OSR) domain, is a negative regulator of grain size and organ size (Mao et al., 2010). Many previous studies have confirmed that the *GS3* gene can regulate the grain size of rice (Fan et al., 2006; Takano-Kai et al., 2009, 2013; Mao et al., 2010; Nan et al., 2018). Moreover, the *GS3* gene also has a similar function in wheat, and its wheat ortholog *TaGS-D1* has been reported to be associated with grain weight and grain length in wheat (Zhang et al., 2014). Therefore, we concluded that HORVU7Hr1G001910 is a reliable candidate gene to regulate the grain size or weight of barley. Similarly, the traits regulated by other potential candidate genes also showed phenotypes consistent or partially consistent with the traits contained in their corresponding QTL hotspots. These findings might imply that grain size/weight genes of other cereal species have same or similar functions in barley. These barley orthologs of grain size/weight related genes identified from rice, maize, and wheat in our study, provide promising candidate genes for barley grain size and weight.

## Conclusion

In summary, in this study, we identified 60 reliable QTLs and 27 QTL hotspots for the traits of grain size and weight in barley, using a single-environment QTL analysis, MET analysis, and covariate QTL analysis. Moreover, we also systematically explored the genetic correspondence between the QTLs identified in this study and known yield-related genes in barley and grain size/weight related genes in other cereal species. A total of 45 barley genes or orthologs were identified as promising candidate genes for barley grain size and weight, 20 of which were located in the QTL hotspot region underlying barley grain size and weight. These potential candidate genes are worth exploring and studying in detail. Our findings will enhance our understanding of the genetic basis of barley grain size and weight and may provide new information to facilitate high-yield breeding in barley.

## Author Contributions

DS and GS conceived this study and designed the experiments. QW, BD, YC, and YW conducted the experiments and phenotyping measurements. QW performed the statistical analysis and wrote the manuscript. XR coordinated the experiments and oversaw the data analysis. DS and GS modified the manuscript. CL produced the Huaai 11 and Huadamai 6 DH population. All authors read and approved the final version of the manuscript.

## Conflict of Interest Statement

The authors declare that the research was conducted in the absence of any commercial or financial relationships that could be construed as a potential conflict of interest.

## Footnotes

1 http://plants.ensembl.org/

2 http://floresta.eead.csic.es/barleymap
