# Supplementary figures and images for: Dissecting the Genetic Basis of Grain Size and Weight in Barley (Hordeum vulgare L.) by QTL and Comparative Genetic Analyses
Source: Front Plant Sci. 2019 Apr 24;10:469. doi: 10.3389/fpls.2019.00469 (PMC6491919; doi:10.3389/fpls.2019.00469)

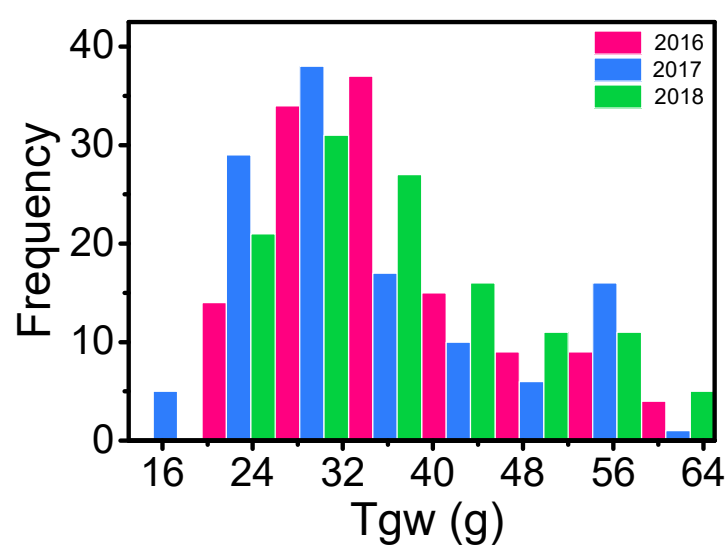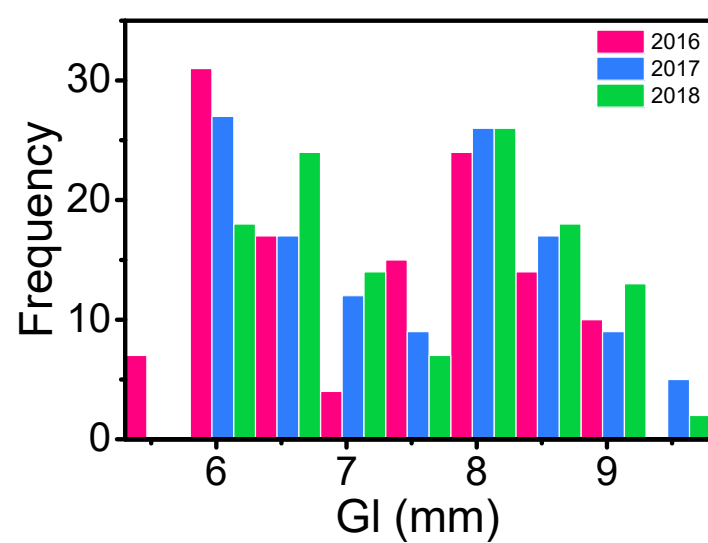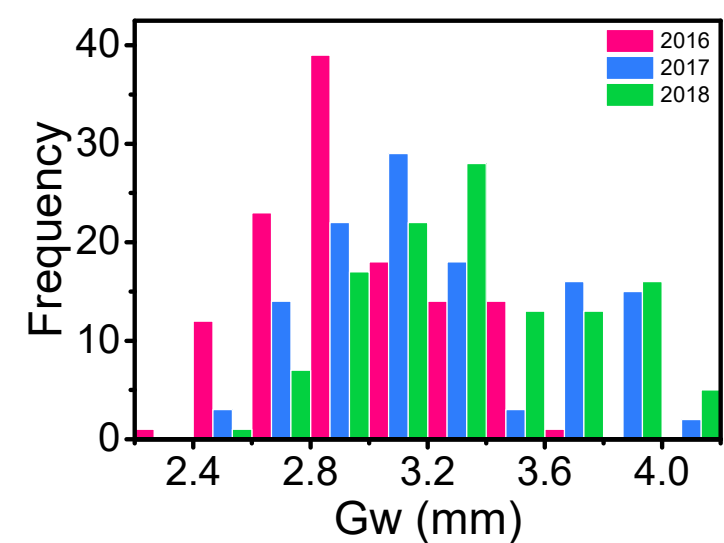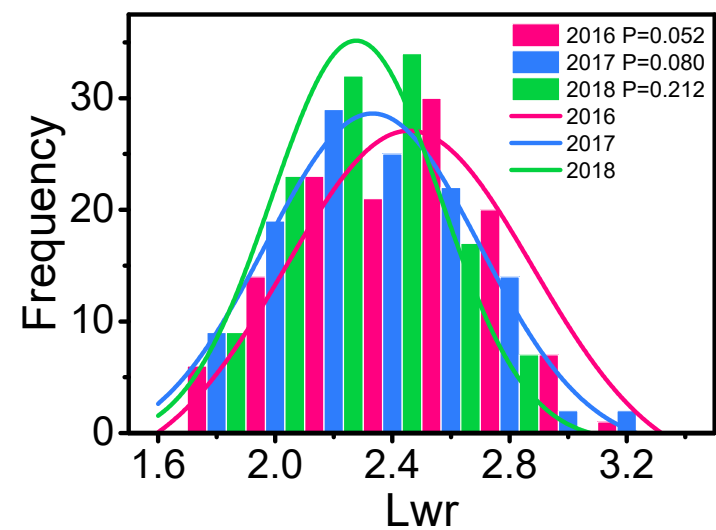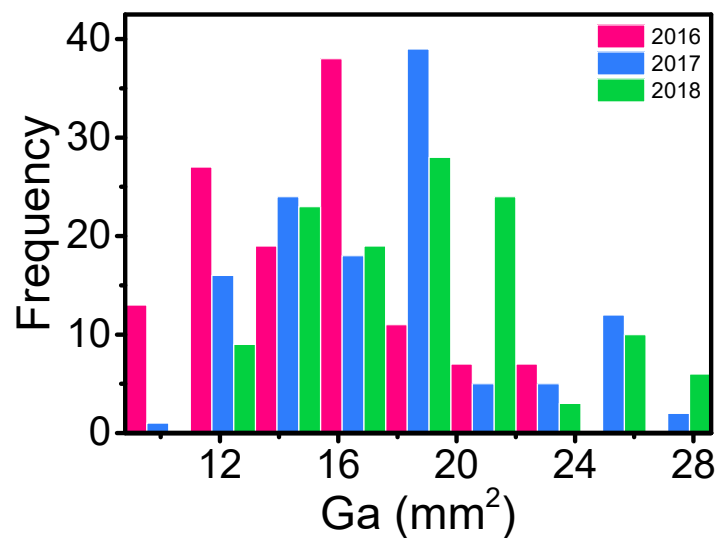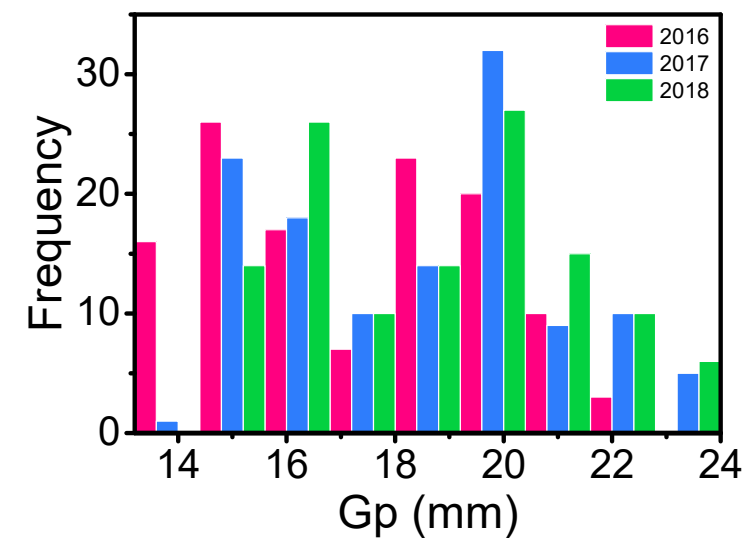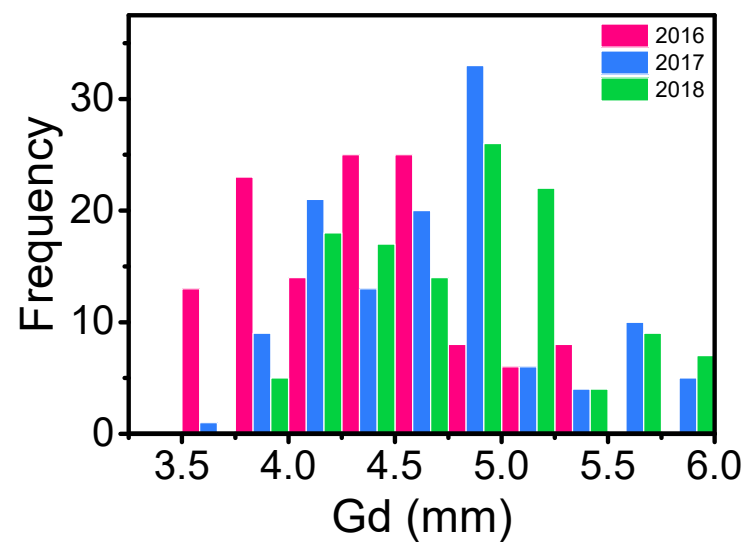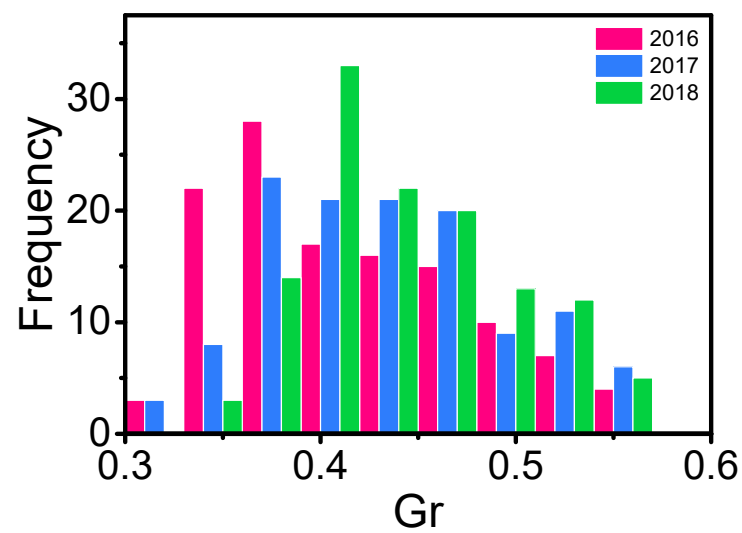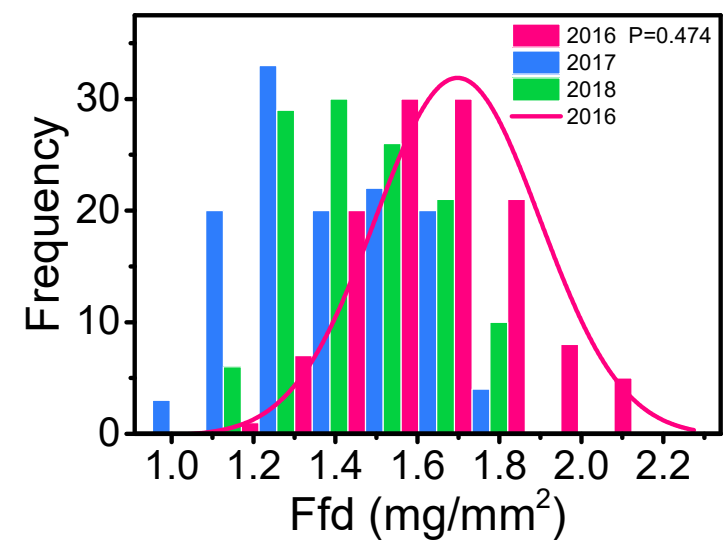

Supplement: Supplementary file 1 [file Image_1.pdf]

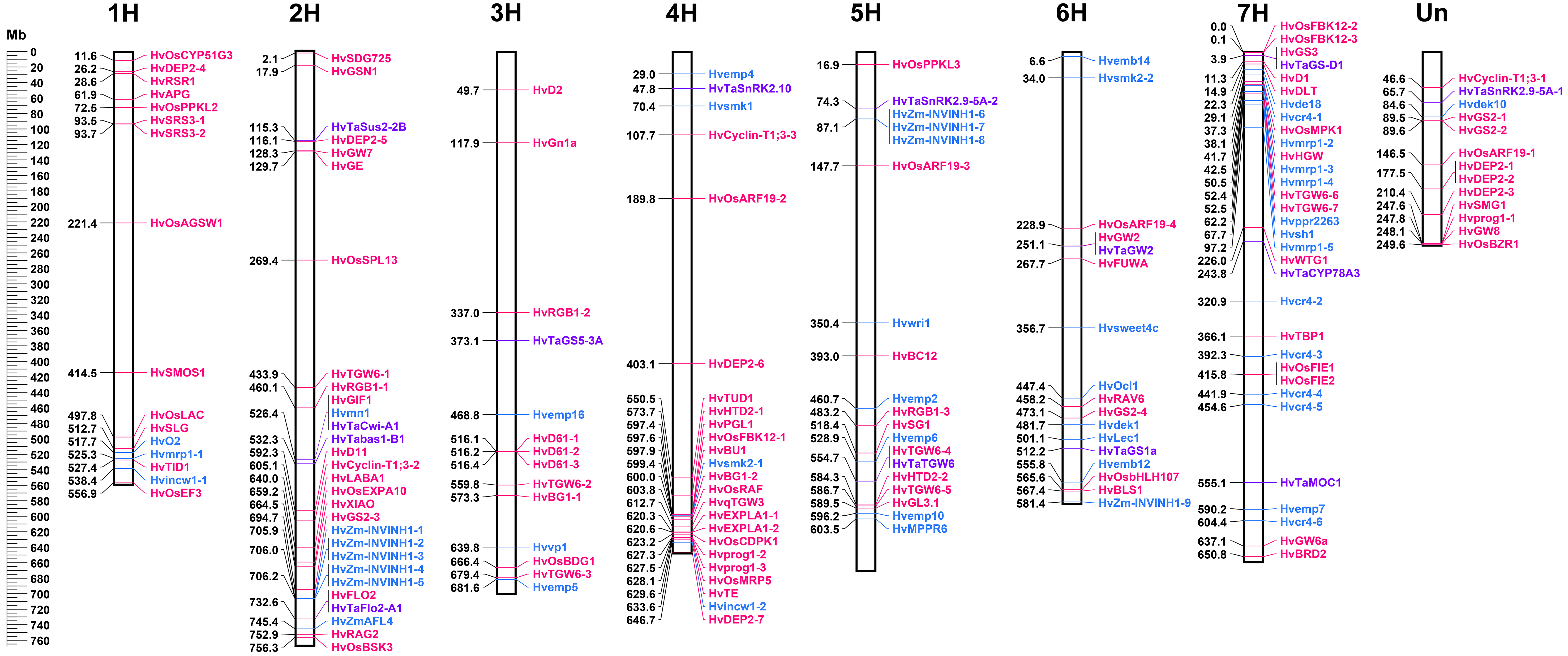

Supplement: Supplementary file 2 [file Image_2.JPEG]
