# Supplementary material for: Dissecting the Genetic Basis of Grain Size and Weight in Barley (Hordeum vulgare L.) by QTL and Comparative Genetic Analyses
Source: Front Plant Sci. 2019 Apr 24;10:469. doi: 10.3389/fpls.2019.00469 (PMC6491919; doi:10.3389/fpls.2019.00469)
Supplement: Supplementary file 4 [file Presentation_1.zip › Supplementary Material.docx]

Supplementary Material

**Dissecting the genetic basis of grain size and weight in barley (*Hordeum vulgare* L.) by QTL and comparative genetic analyses**

Qifei Wang^1^, Genlou Sun^2^, Xifeng Ren^1^, Binbin Du^1^, Yun Cheng^1^, Yixiang Wang^1^, Chengdao Li^3^, Dongfa Sun^1,4*^

^1^College of Plant Science and Technology, Huazhong Agricultural University, Wuhan, China

^2^Biology Department, Saint Mary’s University, Halifax, NS, Canada

^3^School of Veterinary and Life Sciences, Murdoch University, Murdoch, WA, Australia

^4^Hubei Collaborative Innovation Centre for Grain Industry, Yangtze University, Jingzhou, China

*** Correspondence:**Dongfa Sun
College of Plant Science and Technology

Huazhong Agricultural University

Wuhan 430070, China

[sundongfa1@mail.hzau.edu.cn](mailto:sundongfa1@mail.hzau.edu.cn)

**Figure S1. Frequency distribution of nine grain size and weight traits in DH population.** The P value of Shapiro-Wilk test for each year and the trend lines of the accepted normal distribution (*P*>0.05) were shown.

**Figure S2. Chromosome locations of barley orthologs of grain size or weight genes from rice, wheat, and maize in the barley reference genome.** A total of 102 rice orthologs (shown in red), 14 wheat orthologs (shown in blueviolet), and 48 maize orthologs (shown in dodgerblue) were shown.

**Table S1.** QTLs for barley grain size and weight identified in the DH populations using single-environment QTL analysis.

**Table S2.** Stable QTLs identified for nine grain size and weight traits in two or more year using single-environment QTL analysis.

**Table S3.** List of putative pleiotropy or linkage of QTLs detected in different traits by single-environment QTL analysis.

**Table S4.** Putative QTLs for barley grain size and weight identified in the DH populations using multi-environment trait (MET) analysis.

**Table S5.** List of putative pleiotropy or linkage of QTLs detected in different traits by multi-environment trait (MET) analysis.

**Table S6.** QTLs for barley grain size and weight identified in the DH populations using covariate QTL analysis.

**Table S7.** Correspondence between QTLs identified in this study and known barley yield-related genes.

**Table S8.** Genetic correspondence between QTL of barley grain size and weight and other cereal genes affecting grain development.

**Table S9.** Predicted genes in *btwd1* region.
